# aMplitude spectral area of ventricular fibrillation and amiOdarone Study in patients with out-of-hospital cArdIaC arrest. The MOSAIC study

**DOI:** 10.3389/fcvm.2023.1179815

**Published:** 2023-05-15

**Authors:** Francesca Romana Gentile, Lars Wik, Elisabete Aramendi, Enrico Baldi, Iraia Isasi, Jon Erik Steen-Hansen, Sara Compagnoni, Alessandro Fasolino, Enrico Contri, Alessandra Palo, Roberto Primi, Sara Bendotti, Alessia Currao, Simone Savastano

**Affiliations:** ^1^ Division of Cardiology, Fondazione IRCCS Policlinico San Matteo, Pavia, Italy; ^2^ Department of Molecular Medicine, University of Pavia, Pavia, Italy; ^3^ Oslo University Hospital, Division of Prehospital Emergency Medicine, National Service of Competence for Prehospital Acute Medicine (NAKOS), Ullevål Hospital, Oslo, Norway; ^4^ Oslo University Hospital HF, Ullevål Hospital, Oslo, Norway; ^5^ BioRes Group, University of the Basque Country, Bilbao, Spain; ^6^ Division of Prehospital Care, Vestfold Hospital Trust, Tønsberg, Norway; ^7^ AAT 118 Pavia, Agenzia Regionale Urgenza Emergenza at Fondazione IRCCS Policlinico San Matteo, Pavia, Italy

**Keywords:** cardiac arrest, AMSA, ventricular fibrillation, defibrillation, amidoarone

## Abstract

**Objective:**

Antiarrhythmic drugs are recommended for out of hospital cardiac arrest (OHCA) with shock-refractory ventricular fibrillation (VF). Amplitude Spectral Area (AMSA) of VF is a quantitative waveform measure that describes the amplitude-weighted mean frequency of VF, it correlates with intramyocardial adenosine triphosphate (ATP) concentration, it is a predictor of shock efficacy and an emerging indicator to guide defibrillation and resuscitation efforts. How AMSA might be influenced by amiodarone administration is unknown.

**Methods:**

In this international multicentre observational study, all OHCAs receiving at least one shock were included. AMSA values were calculated by retrospectively analysing the pre-shock ECG interval of 2 s. Multivariable models were run and a propensity score based on the probability of receiving amiodarone was created to compare two randomly matched samples.

**Results:**

2,077 shocks were included: 1,407 in the amiodarone group and 670 in the non-amiodarone group. AMSA values were lower in the amiodarone group [8.8 (6–12.7) mV·Hz vs. 9.8 (6–14) mV·Hz, *p* = 0.035]. In two randomly matched propensity score-based groups of 261 shocks, AMSA was lower in the amiodarone group [8.2 (5.8–13.5) mV·Hz vs. 9.6 (5.6–11.6), *p* = 0.042]. AMSA was a predictor of shock success in both groups but the predictive power was lower in the amiodarone group [Area Under the Curve (AUC) non-amiodarone group 0.812, 95%CI: 0.78–0.841 vs. AUC amiodarone group 0.706, 95%CI: 0.68–0.73; *p* < 0.001].

**Conclusions:**

Amiodarone administration was independently associated with the probability of recording lower values of AMSA. In patients who have received amiodarone during cardiac arrest the predictive value of AMSA for shock success is significantly lower, but still statistically significant.

## 1. Introduction

Ventricular Fibrillation (VF) is one of the rhythms in adult out-of-hospital cardiac arrest (OHCA) (1). Correct treatments are prompt defibrillation and cardiopulmonary resuscitation (CPR) (2, 3). Data supporting the use of antiarrhythmic drugs after three ineffective shocks is sparse (4). Their effects on improving the rate of return of spontaneous circulation (ROSC) and survival to hospital admission are weak (5, 6). None of them has shown increased long-term or survival to discharge with good neurological outcomes. Amiodarone may improve short-term outcome (ROSC and survival at hospital admission) (7, 8), but this might be effective only for shock-refractory VF/pulseless ventricular tachycardia (pVT) in bystander-witnessed arrests (9).

The Amplitude Spectral Area (AMSA) of VF is a quantitative waveform measure that describes the amplitude-weighted mean frequency of VF. In animal studies AMSA correlates with intramyocardial adenosine triphosphate (ATP) concentration levels (10) and with coronary perfusion pressure (11). Therefore it has been proposed as a tool to monitor the effectiveness of chest compressions (12). The AMSA values can be influenced by the quality of CPR, different myocardial substrates (13, 14) and patient characteristics (15). Interestingly, it was highlighted that drugs, such as beta-blockers (16), may also alter AMSA. Amiodarone is largely used during resuscitation for unresponsive defibrillation of VF/pVT but no studies have determined if its administration is able to affect AMSA or the myocardium during CPR.

It has been demonstrated that higher AMSA values are associated with higher shock success and ROSC (15, 17). AMSA-driven shocks and epinephrine administration resulted in less post-resuscitation myocardial dysfunction and better survival (18). Because AMSA may predict if defibrillation could terminate VF with concurrent ROSC, AMSA was proposed as a tool to guide defibrillation in adults (17). However, it's unknown whether amiodarone may alter the predictive power of AMSA and consequently AMSA's clinical use.

We sought to determine if OHCA patients who received amiodarone during advanced cardiac life support (ACLS) had lower values of AMSA compared to those who did not receive amiodarone. Secondly, we wanted to examine whether the rates of successful defibrillation, ROSC and survived event would differ between the amiodarone and non-amiodarone groups. Finally, we wanted to assess if the role of AMSA as a predictor of shock success is maintained both in the amiodarone group and in the non-amiodarone group.

## 2. Material and methods

### 2.1. Type of study and population

This is a multicentre observational study based on retrospective analysis of prospectively collected data (ClinicalTrials.gov Identifier: NCT04997980). All OHCAs occurring between January 1, 2015, and December 31, 2020, in the province of Pavia (Italy) and between January 1, 2007, and December 31, 2018 in Vestfold county (Norway) were considered. If at least one shock for VF during ACLS was delivered, regardless of whether the first rhythm was shockable or not, the patient was eligible for inclusion. Data were retrieved from the Lombardia CARe Registry for the province of Pavia, and from the Vestfold Cardiac Arrest Registry for the region of Vestfold which are described in the Supplementary materials.

### 2.2. Data collection and analysis

Anonymized data from the two different databases were integrated and combined in a single *ad hoc* database for statistical analysis (see Supplementary materials). After the electronic data of all cases had been extracted from the monitor/defibrillators' memories (Corpuls 3 for the province of Pavia and LIFEPAK 12/15 monitors Vestfold), ECG signals were processed by Matlab software (The MathWorks, Inc., Natick, USA). Only OHCA patients who had at least one manual defibrillation attempt were considered. All shocks were independently reviewed by three cardiologists from our team and annotated as successful/unsuccessful shocks. Based on the lack of a uniform definition of shock success in literature (19) and consistent with our previous work (20) we have defined successful defibrillation as the cessation of VF or pVT with the subsequent emergence of an organized rhythm within 60 s. An organized rhythm required at least two QRS complexes separated by no more than 5 s each.

For every shock, AMSA was computed using a 2 s pre-shock ECG interval, free of chest compression artifacts, leaving a 1s guard before the shock. The ECG was bandpass filtered (0.5–30 Hz) using a forward-backward order 8 elliptic filter to remove baseline oscillations and high frequency noise. Fast Fourier Transform was used to compute the spectral amplitudes of the ECG, and AMSA was calculated in the 2–48 Hz frequency range (15).

For each patient, all pre-hospital variables were included according to the 2014 Utstein recommendations (21). ROSC was annotated by clinicians on scene after every shock. ROSC was assumed, even if transient, in the presence of a palpable pulse checked according to guidelines (2, 3).

Following international recommendations (2, 3) amiodarone was administered either via an intravenous or an intraosseous line at the dosage of 300 mg for the first bolus followed by an additional dose of 150 mg.

### 2.3. Statistical analysis

Categorical variables were compared with the Chi-square test and presented as number and percentage. Continuous variables were compared with the *t*-test and presented as mean ± standard deviation or compared with the Mann–Whitney test and presented as median and interquartile range (IQR) for normal distributions (tested with the D'Agostino-Pearson test). Uni- or multivariable logistic regression were applied to assess the association between one binomial dependent variable and one or more not correlated independent variables.

In a per-shock analysis, the values of AMSA preceding shocks delivered to patients treated with amiodarone were compared with the values of AMSA preceding shocks delivered to patients not treated with amiodarone.

The same analysis was performed by a propensity score matching analysis. The propensity score was created based on the coefficients resulting from a multivariable logistic regression model for the probability of receiving amiodarone considering age, sex, the presence of bystander CPR, the call to shock time for every single shock, the use of mechanical CPR, the administration of dispatcher assisted CPR, the year and study site (Pavia or Vestfold) as independent variables. Once created, the propensity score was tested for linear prediction. A pool of shocks with a similar propensity score was identified and then, for each case in the amiodarone group, a control in the non-amiodarone group was randomly assigned.

The shock success prediction accuracy of AMSA was tested using the receiver operating characteristic (ROC) curve analysis. After the creation of the curve, by plotting for each value of AMSA the true positive rate (shock success in case of expected shock success) in function of false positive rate (shock failure in case of expected shock success) the area under the curve (AUC) was calculated according to the Hanley and McNeil methodology. The comparison the ROC curve was run according to the DeLong method.

## 3. Results

### 3.1. Study population characteristics

A total of 629 EMS-assessed OHCAs were enrolled in the study: 250 from Pavia and 379 from Vestfold. Table 1 shows the main characteristics of the population.

**Table 1:** Patients’ characteristics.

Variable	Overall (*N* = 629)
Study site (%)	
Pavia	250 (40)
Vestfold	379 (60)
Age (IQR) (years)	68 (57–77)
Male gender (%)	480 (78)
EMS arrival time (IQR) (min)	9.5 (6.9–13.4)
Medical aetiology (%)	564 (90)
OHCA location (%)	
Home	414 (66)
Nursing home	6 (1)
Street	112 (18)
Public building	21 (3)
Workplace	17 (2.5)
Sport	4 (1)
Other	37 (6)
Unknown	18 (2.5)
Telephone CPR (%)	316 (50)
Witnessed event (%)	
No	112 (18)
EMS	68 (11)
Bystanders	425 (68)
Unknown	24 (3)
Bystander CPR (%)^a^	409 (76)
Shockable presenting rhythm (%)	397 (67)
AED Use before EMS arrival (%)^a^	67 (12)
Number of shocks delivered (IQR)	3 (1–6)
Amiodarone (%)	
Yes	253 (40)
No	347 (55)
Unknown	29 (5)
Amiodarone administered with <3 shocks (%)^b^	23 (9)
Amiodarone administered with ≤3 shocks (%)^b^	56 (22)
Amiodarone not administered with more than 3 shocks (%)^c^	64 (18.4)
Mechanical CPR (%)	389 (64)
ROSC (%)	267 (42)
Survived event (%)	230 (37)

^a^ EMS Witnessed excluded.

^b^ Only patients treated with amiodarone considered.

^c^ Patients treated with amiodarone excluded.

By comparing two random samples (120 patients form Pavia and 120 patients from Vestfold), homogeneous for sex, number of shocks received, age and call to shock time, the AMSA values were similar in the two study sites [Pavia: 8.3 (5.1–10.9) mV·Hz vs. Vestfold: 9.4 (4.9–14.5) mV·Hz, *p* = 0.11]. Moreover, AMSA values were found to predict shock success in both regions' study groups with no statistical difference at the Receiver operating characteristic (ROC) curve analysis (AUC Pavia 0.786, 95%CI: 0.756–0.813; AUC Vestfold 0.759, 95%CI: 0.735–0.782; *p* = 0.206) Supplementary Figure S1.

Out of the entire population, 253 patients received amiodarone and 347 did not (29 patients data unknown). The amiodarone group had a higher percentage of males, of medical aetiology and of witnessed events. The number of shocks delivered were higher in the amiodarone group, as well as the frequency of both telephone and mechanical CPR. However, the trends of ROSC and survived event percentages were lower in the amiodarone group compared to the non-amiodarone group. Other patients' characteristics are presented in Table 2.

**Table 2:** Patients’ characteristics in amiodarone and non-amiodarone groups.

Variable	Amiodarone (*N* = 253)	Non-Amiodarone (*N* = 347)	*p*-value
Age (IQR) (years)	67 (56–76)	69 (58–78)	0.12
Male gender (%)	212 (84)	250 (72)	<0.001
EMS arrival time (IQR) (min)	9.6 (7–14)	9.5 (7–13)	0.56
Medical aetiology (%)	238 (94)	302 (87)	0.005
OHCA location (%)			0.49
Home	165 (65)	233 (67)	
Nursing home	1 (0)	5 (1)	
Street	49 (19)	53 (15)	
Public building	6 (2)	15 (4)	
Workplace	6 (2)	11 (3)	
Sport	1 (0)	3 (1)	
Other	16 (6)	19 (5)	
Unknown	9 (4)	8 (2)	
Telephone CPR (%)	141 (56)	160 (46)	0.01
Witnessed event (%)			0.005
No	40 (16)	68 (20)	
EMS	17 (7)	48 (14)	
Bystanders	187 (74)	219 (63)	
Unknown	9 (3)	12 (3)	
Bystander CPR (%)^a^	178 (78)	211 (74)	0.19
Shockable presenting rhythm (%)	194 (73)	187 (54)	<0.001
AED Use before EMS arrival (%)^a^	22 (10)	39 (14)	0.13
Number of shocks delivered (IQR)	6 (4–8)	2 (1–3)	<0.001
Mechanical CPR (%)	182 (72)	191 (55)	<0.001
Epinephrine (mg) (IQR)	5 (4–7)	4 (2–5)	<0.01
ROSC (%)	98 (39)	152 (44)	0.15
Survived event	87 (34)	127 (37)	0.51
AMSA at first shock median (IQR) (Hz·mV)	9.8 (7–13)	9.7 (6–15)	0.9

EMS, emergency medical service; CPR, cardiopulmonary resuscitation; AED, Automated external defibrillator.

^a^ EMS witnessed excluded.

### 3.2. Shock characteristics based on amiodarone administration

The total number of shocks, 2,077 for the 600 OHCA patients, were divided into patients with and without amiodarone administered. In the amiodarone group shock success rate was lower than in the non-amiodarone group. The AMSA values were also lower in the amiodarone group (Table 3).

**Table 3:** Shocks characteristics in amiodarone and non-amiodarone groups.

Shocks characteristics (*N* = 2,077)	Amiodarone (*N* = 1,407)	Non-Amiodarone (*N* = 670)	*p*-value
Energy delivered (IQR) (J)	300 (200–360)	200 (150–200)	<0.001
Pavia (Corpuls)	200 (150–200)	150 (150–200)	<0.001
Vestfold (Lifepak)	360 (300–360)	200 (200–300)	<0.001
Successful (%)	463 (33)	278 (41)	<0.001
AMSA (IQR) (Hz·mV)	8.8 (6–13)	9.8 (6–14)	0.035

### 3.3. Primary outcome

#### 3.3.1. AMSA values according to amiodarone administration

**Figure 1:**
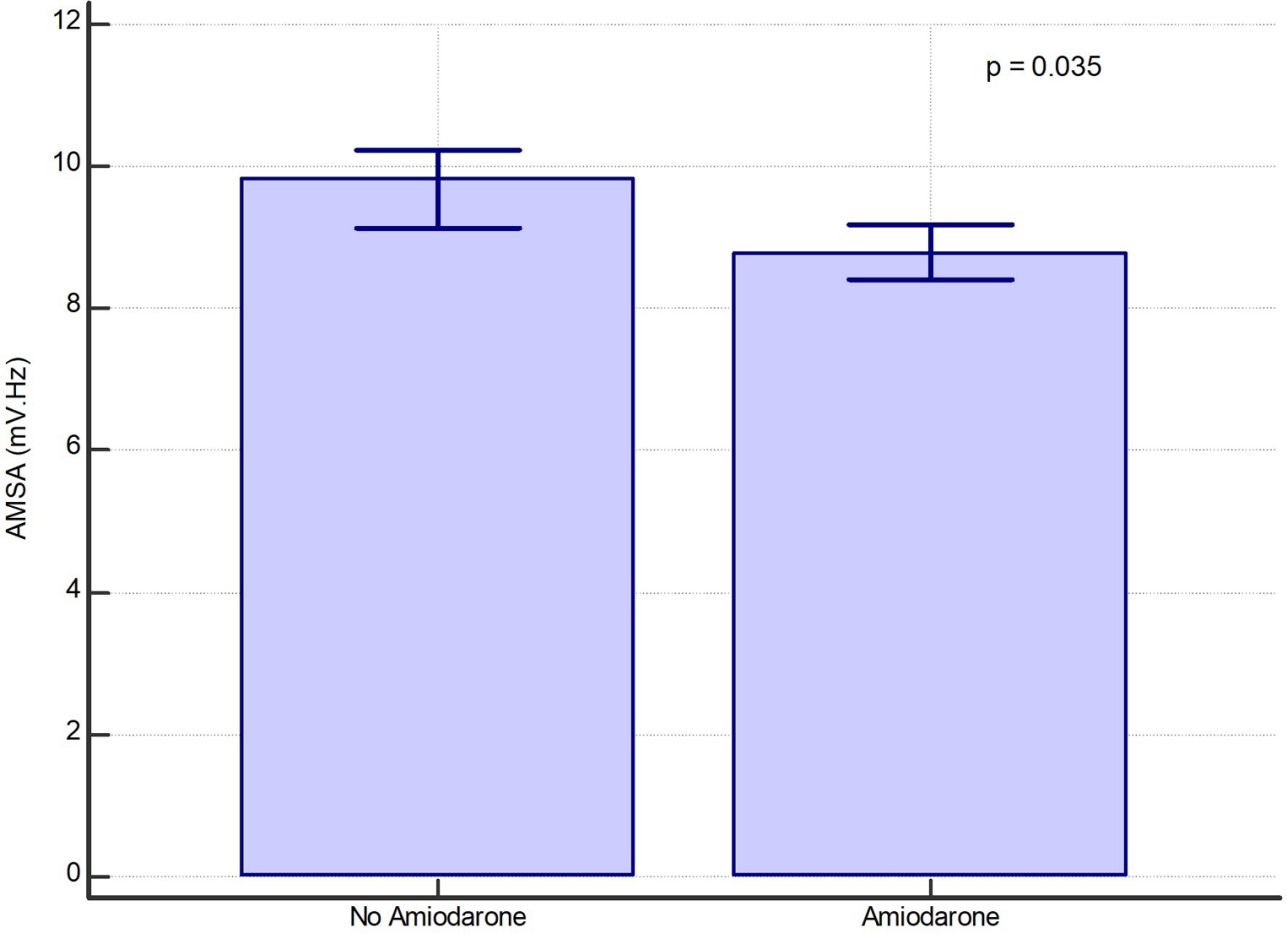
Bar graph of median values of AMSA with their 95% confidence interval in the amiodarone and in the non-amiodarone groups in the whole population of shocks.

**Figure 2:**
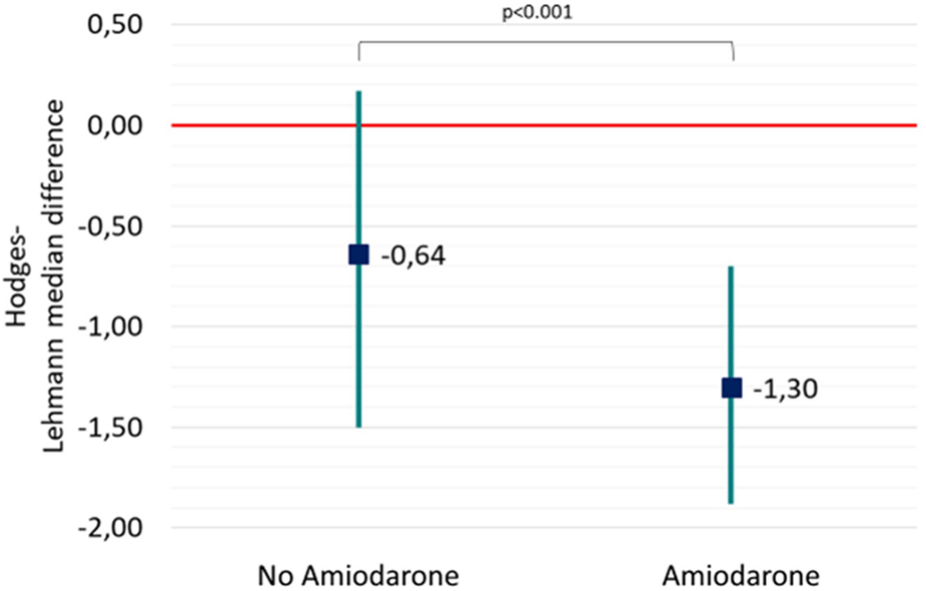
Hodges-Lehmann median difference and 95% confidence showing the reduction of AMSA values from the first two shock to the successive ones both in the non-amiodarone and in the amiodarone group.

In a per-shock analysis, AMSA values were significantly lower in the group of shocks delivered to patients treated with amiodarone [8.8 (6–12.7) mV·Hz vs. 9.8 (6–14) mV·Hz, *p* = 0.035] (Figure 1). In the non-amiodarone group, the reduction of AMSA values from the first two shocks to the successive ones was not statistically significant [10 mV·Hz (5.9–17.4) vs. 9.1 mV·Hz (5.8–12.8), *p* = 0.123]. On the contrary, in the amiodarone group AMSA decreased significantly after the second shock [10.2 mV·Hz (6.6–14.2) vs. 8.3 mV·Hz (5.8–12.2), *p* < 0.01]. Therefore, the extent of the reduction of AMSA after the second shock was greater in the amiodarone group [−1.3 (−1.9; −0.7) vs. −0.6 (−1.5; 0.2), *p* < 0.001] (Figure 2).

**Figure 3:**
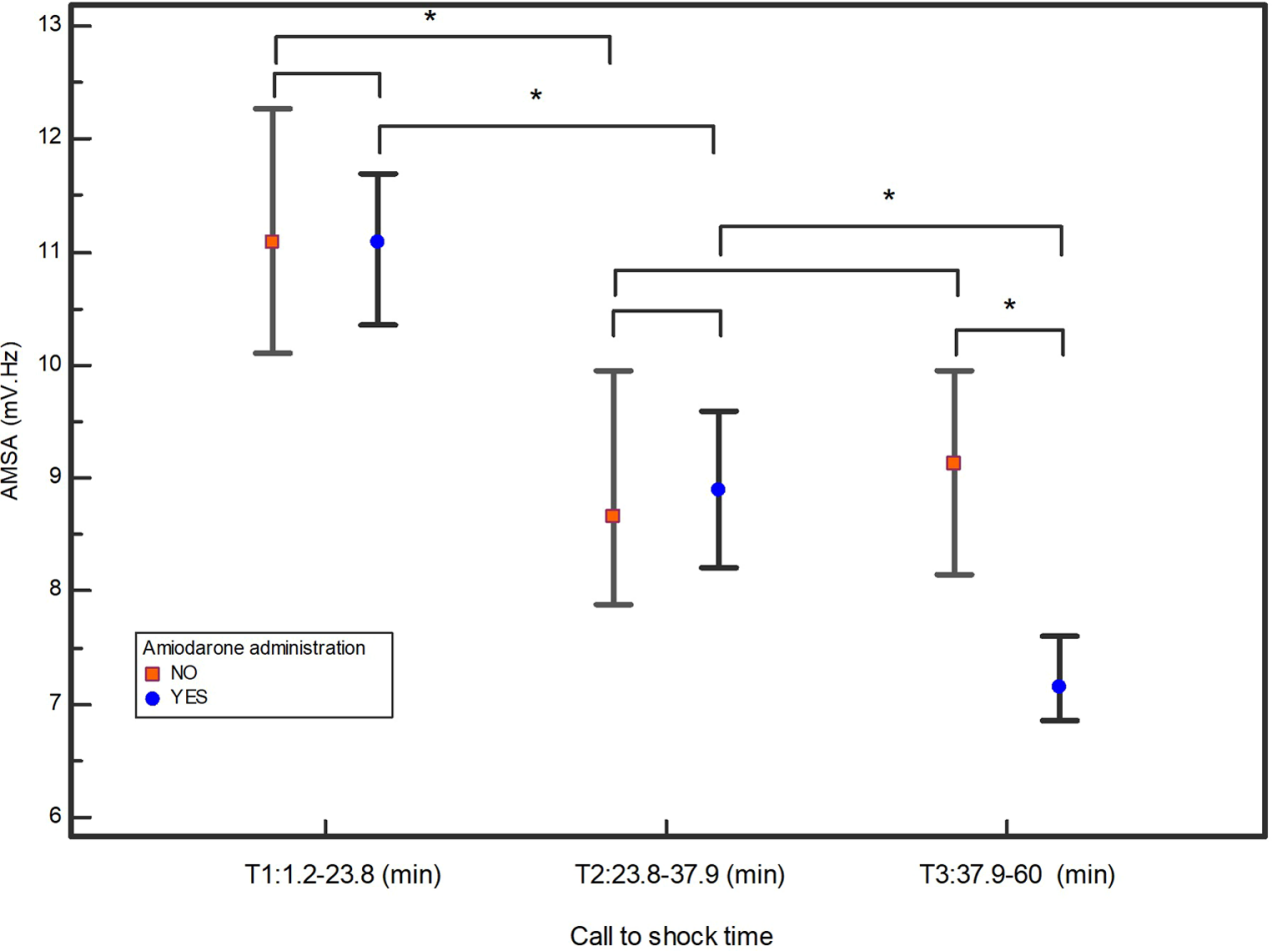
Median values of AMSA and their 95% confidential interval in the three tertiles of call to shock time. *indicates statistically significant differences.

By plotting the median AMSA values of the amiodarone and non-amiodarone groups in each of the three tertiles based on the call to shock time, the amiodarone group showed a statistically significant reduction in AMSA between T1 and T2 and between T2 and T3. Conversely, in the non-amiodarone group there was a significant reduction only between T1 and T2 (Figure 3).

In the multivariable logistic regression analysis corrected for age, bystander CPR, witnessed event, year 2020, call to shock time, shockable presenting rhythm, shock energy, multiple shocks, sex and study site (Pavia and Vestfold), the treatment with amiodarone was independently associated with AMSA values lower than the median (9.4 mV·Hz) [OR 1.33, (95%CI: 1.1–1.6), *p* = 0.009].

**Figure 4:**
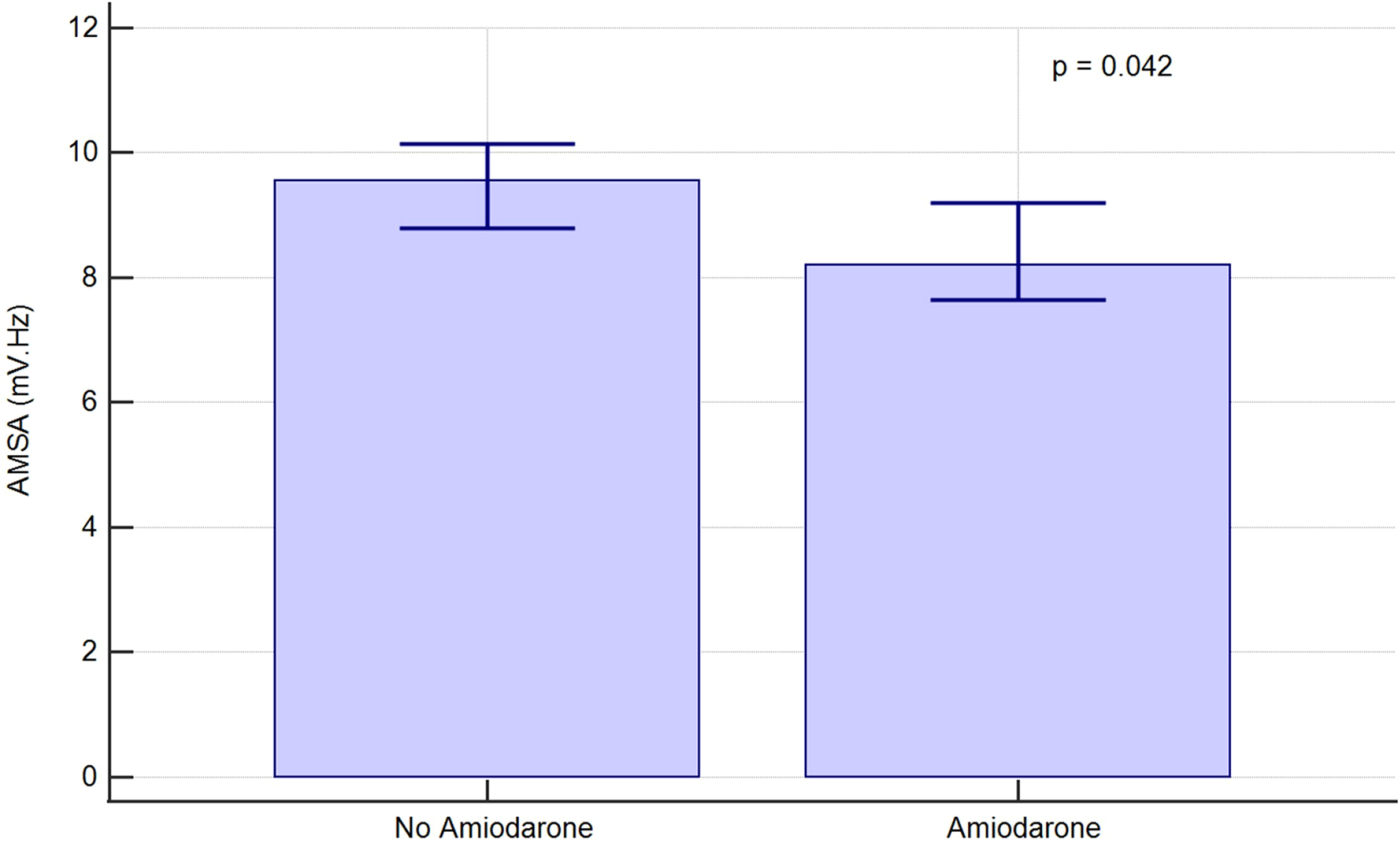
AMSA median values and 95% confidence interval in randomly matched, propensity score-based groups of shocks.

AMSA values were then compared in two randomly matched propensity score-based groups of 261 shocks each. The covariates inserted in the model and the resulting coefficients are shown in Supplementary Table S1. AMSA was again demonstrated to be lower in the amiodarone group [8.2 (5.8–13.5) mV·Hz vs. 9.6 (5.6–11.6), *p* = 0.042] as shown in Figure 4.

**Figure 5:**
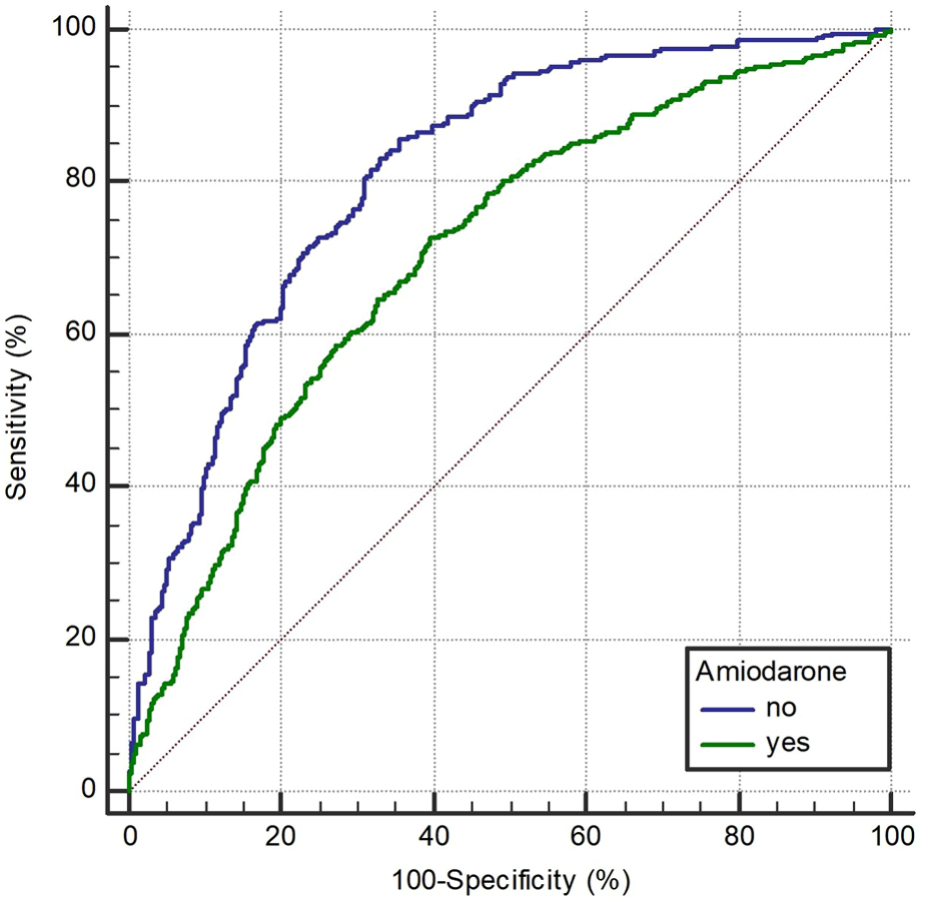
Receiver operating characteristic curve of AMSA for the prediction of shock success in amiodarone and non-amiodarone group.

### 3.4. Secondary outcomes

#### 3.4.1. Shock success, ROSC and survived event rates

By comparing the amiodarone and the non-amiodarone randomly matched groups based on the propensity score analysis, the shock success rate did not statistically differ (non-amiodarone 38% vs. amiodarone 36%, *p* = 0.6). After correction for age, sex, EMS arrival time, the presence of bystander CPR, the presence of a shockable presenting rhythm, the number of shocks received, the study site and the first available AMSA value, the treatment with amiodarone did not influence the probability of both ROSC [OR 0.8 (95%CI: 0.4–1.4), *p* = 0.38] and survival [OR 0.8 (95%CI: 0.4–1.5), *p* = 0.46].

#### 3.4.2. AMSA as a shock success predictor

In the ROC curve analysis (Figure 5), AMSA values were found to be able to predict shock success in both the amiodarone and the non-amiodarone groups, however the predictive power was significantly lower in the amiodarone group (AUC 0.812, 95%CI: 0.78–0.841 vs. 0.706, 95%CI: 0.68–0.73; *p* < 0.0001).

## 4. Discussion

Amiodarone is extensively used during resuscitation for unresponsive defibrillation of VF/pVT but very little is known about how and to what extent administration of intravenous amiodarone may affect VF. The main finding of this study was that the values of AMSA which quantitatively measure the VF waveform, in the amiodarone group were lower than in the non-amiodarone group. In fact, the values of the first shocks, prior to the administration of amiodarone, were similar in the two groups while the reduction of AMSA at the successive shocks was more pronounced in the amiodarone group. In the amiodarone group there was an almost linear reduction of AMSA over time. This is in contrast to the non amiodarone group, in which the decline of AMSA values was not evident, as if amiodarone had hastened the decrease of amplitude of VF.

We might argue that the decreased values of AMSA in the amiodarone group could be explained by a longer resuscitation and a higher number of shocks. However, we found that amiodarone was independently associated with the probability of recording lower values of AMSA even after correction for all the OHCA characteristics known (or potentially able) to affect the patient's outcome, such as time to each shock, sex, age, witnessed event, bystander CPR, study site (Pavia and Vestfold) and year 2020. We adjusted our analysis for sex because it was suggested that males had lower AMSA than females (15). Time to shock and bystander CPR play a confounding role because longer resuscitation time leads to a greater loss of ATP in myocardiocytes which would be reflected by lower AMSA values (10). Finally, we corrected for the year 2020, which led to prolonged EMS response time due to the COVID-19 pandemic (22).

The hypothesis that antiarrhythmic effect of drugs on the myocardium would be quantifiable through the analysis of electrocardiograms was proposed ten years ago by Sherman et al. (16). This topic was also indirectly approached regarding the effect of lidocaine and amiodarone on quantitative ECG waveform measures in a recent sub-analysis from the clinical ROC-ALPS study by Salcido et al. (23). However, none of these types of quantification have had practical repercussions on resuscitation.

Amiodarone has predominantly a Vaughan-Williams class-III effect of potassium channel blockade resulting in lengthening of the cardiac action potential, together with a class I use-dependent sodium channel blockade of inward sodium currents, a class II beta receptor blockade and class IV calcium channel blockade (24). The consequent increased refractoriness of cardiac tissue and the slowed ventricular conduction are thought to facilitate successful defibrillation and to reduce the risk of recurrent arrhythmias (25). The complex pharmacologic profile of amiodarone as well as the heterogeneity of underlying VF mechanisms make this query very challenging. Animal studies that have focused on the ionic and cellular mechanisms of amiodarone use or changes in the defibrillation threshold due to the acute administration of the drug (26–28) have been somewhat contradictory. The rather modest evidence coming from human-based randomized trials and metanalyses (7–9) together with the limited existing therapeutic options in resuscitation have led to the adoption of amiodarone as the preferential treatment of life-threatening ventricular tachyarrhythmias.

Previous studies have suggested a marginal effect of cardiac medications on AMSA values (29, 30). However, that conclusion was drawn considering only oral chronic intake. In the paper by Hulleman and colleagues class III and I antiarrhythmic drugs were considered together and they found halved AMSA values even if with a non-statistically significant *p* value of 0.069 probably due to the small number of patients treated (only 1.8%). Conversely, the present study was focused on the acute effect of intravenous amiodarone. The administration route is accompanied by substantial differences; In fact, it has been shown how the oral and the intravenous administration were different due to the effects mediated by the active metabolite desethylamiodarone (DEA) resulting from the first-pass hepatic metabolism (28).

The underlying cause of cardiac arrest was also shown to affect AMSA values. Olasveengen and colleagues (31) found that patients with an acute myocardial infarction had lower AMSA values as compared to other cardiac arrest aetiology. Although we don't know the definite cause of cardiac arrest however an acute coronary syndrome is by far the most frequent cause of adult cardiac arrest (32) and it is included in the Utstein category named “medical aetiology” which accounted for about ninety percent and was higher in the amiodarone group.

Due to the observational nature of this study, the decision to administer amiodarone was not randomized. In Pavia the decision was done by the physician and in Vestfold by the paramedic crew. To reduce possible selection bias, we ran a propensity score analysis to compare two independent groups having *a priori* the same probability of receiving amiodarone. This additional analysis showed, once again, that patients treated with amiodarone had significantly lower values of AMSA.

Although this study was not designed for survival analysis, we found that amiodarone administration was not associated with a higher probability of shock success, ROSC or survived event. To our knowledge, no previous study has compared the efficacy of amiodarone in terms of shock success in OHCA patients. Our results regarding ROSC are aligned with the results from the ROC-ALPS trial (9), which randomized more than three thousand patients in three arms of treatment (amiodarone, lidocaine and placebo), finding no difference in terms of ROSC or survival at hospital discharge between amiodarone and placebo. However, the trial found a statistically significant difference in terms of the number of patients admitted to hospital (amiodarone 45.7% vs. placebo 39.7%, *p* = 0.01). In this regard, our results about survived event could seem in contrast with the ROC-ALPS trial at first glance. However, our endpoints are slightly different from that study. We have considered “survived event” according to the most recent Utstein definition that describes it as a ROSC sustained until arrival at the emergency department (ED) and transfer of care to medical staff at the receiving hospital. Instead, the ROC-ALPS used survival at hospital admission as a secondary endpoint. Our endpoint “survived event” does not exactly mirror “survived at hospital admission” because OHCA patients admitted to the hospital with ongoing CPR may still expire prior to achieving ROSC.

The effect of amiodarone could limit the ability of AMSA to predict defibrillation outcomes. This topic is of great clinical importance because AMSA is an emerging indicator that might guide defibrillation and resuscitation efforts. One randomized clinical study, even if terminated early due to low inclusion rates because it was started when the Covid 19 pandemic evolved, showed that the real-time AMSA measuring during resuscitation of OHCA patients is feasible (33). It is of pivotal importance to know if the administration of amiodarone can affect both the values and predictivity of AMSA. Our study found that, even though AMSA remains a shock success predictor in both groups, the area under the curve of the ROC-curve is significantly lower in the amiodarone group. After the administration of amiodarone, the cut-off of AMSA could be different from that at the beginning of ACLS. In a clinical scenario, we speculate that the chances of an error could be greater if defibrillation was guided by AMSA values after the administration of amiodarone. There is therefore a need for a prospective randomized clinical study where amiodarone effect on AMSA value is taken into consideration.

### 4.1. Limitations

This study has some potential limitations. First, it is an observational study with the related intrinsic limitations. Second, we were unable to provide a direct comparison between AMSA values before and after the administration of amiodarone. The main reason for this is that in our two registries, the use of amiodarone is annotated but the exact time of administration is absent as this is not requested by the Utstein template. Because 22% of the patients treated with amiodarone received the drug within the third shock, we considered the first two shock as those most likely to be pre-amiodarone. One possibility for those who received amiodarone earlier than the third shock is that shocks given prior to ACLS (for example during BLS-D or by bystanders with AED) were considered for the purposes of the advanced resuscitation algorithm. We decided to run multivariable model of logistic regression, and a comparison of propensity score-matched group to mitigate this limitation. Third, consistently to the Utstein recommendations, we did not annotate the use of lidocaine. Presumably, some of the patients who did not receive amiodarone were treated with lidocaine; however, the reduction of AMSA from the first two shocks towards the successive shocks was not significant in this group. Fourth, we had no information of patient's home therapies or chronic comorbidities that could affect AMSA, but this is a common limitation for studies based on retrospectively collected Utstein data. Additionally, it was demonstrated by Hulleman et al. that these factors have little impact on AMSA values (29). Fifth, the definite cause of cardiac arrest was not available so we don't know the precise prevalence of acute myocardial infarction in the amiodarone and non-amiodarone group. According to the Utstein style acute myocardial infarction is included in the definition of “medical etiology” which was about ninety percent in both groups.

## 5. Conclusion

The use of amiodarone in advanced resuscitation is associated with lower values of AMSA of VF in patients with out-of-hospital arrest after correcting for patient and OHCA characteristics. Moreover, AMSA maintains its predictive role in shock success in patients who have received amiodarone, although with a significantly lower predictive power compared to patients who did not. We believe that these results will not only help to define AMSA's role and use in resuscitation but also could launch AMSA as an additional data point to better understand the controversial role of amiodarone in cardiac arrest.

## Data Availability

The raw data supporting the conclusions of this article will be made available by the authors, without undue reservation.
