# Supplementary material for: aMplitude spectral area of ventricular fibrillation and amiOdarone Study in patients with out-of-hospital cArdIaC arrest. The MOSAIC study
Source: Front Cardiovasc Med. 2023 May 15;10:1179815. doi: 10.3389/fcvm.2023.1179815 (PMC10226588; doi:10.3389/fcvm.2023.1179815)
Supplement: Supplementary file 1 [file Table1.docx]

**Table 1S. Coefficients for propensity score calculation.**

| Variable | Coefficient | Std. Error | Wald | P value |
| --- | --- | --- | --- | --- |
| Age | -0.00394 | 0.00358 | 1.2088 | 0.2716 |
| Bystander CPR | 0.26664 | 0.14807 | 3.2426 | 0.0717 |
| Call to shock time (min) | 0.00128 | 0.00157 | 0.6633 | 0.4154 |
| Mechanical CPR | 0.66005 | 0.10520 | 3.,3694 | <0.0001 |
| Male Sex | 0.71946 | 0.13396 | 28.8444 | <0.0001 |
| Telephone CPR | 0.14686 | 0.14423 | 1.0369 | 0.3085 |
| Study Site | 0.32851 | 0.14842 | 4.8989 | 0.0269 |
| Year | 0.00378 | 0.01796 | 0.0443 | 0.8334 |
| Constant | -8.46804 | 36.34283 | 0.0543 | 0.8158 |
