# Supplementary material for: aMplitude spectral area of ventricular fibrillation and amiOdarone Study in patients with out-of-hospital cArdIaC arrest. The MOSAIC study
Source: Front Cardiovasc Med. 2023 May 15;10:1179815. doi: 10.3389/fcvm.2023.1179815 (PMC10226588; doi:10.3389/fcvm.2023.1179815)
Supplement: Supplementary file 2 [file Datasheet1.docx]

**Supplementary materials**

**Cardiac Arrest registries**

Both the Lombardia CARe and the Vestfold cardiac arrest registry are two Utstein based registries.

The Lombardia CARe registry is the largest cardiac arrest registry currently existing in Italy. It includes 7 provinces of the Lombardy region (Northern Italy) and covers a population of more than 4 million inhabitants. All the operational and clinical data from pre-hospital to hospital settings, including OHCA patients’ follow-up, are available from 2015 for the Province of Pavia. Data from Corpuls 3 defibrillators (GS Elektromedizinische Geräte G Kaufering, Germany) were stored in the Fondazione IRCCS Policlinico San Matteo in Pavia. The Lombardia Care Registry is using REDCap (Research Electronic Data Capture) hosted at the Fondazione IRCCS Policlinico San Matteo (25,26). REDCap is a secure, web-based application designed to support data capture for research studies, providing 1) an intuitive interface for validated data entry; 2) audit trails for tracking data manipulation and export procedures; 3) automated export procedures for seamless data downloads to common statistical packages; and 4) procedures for importing data from external sources.

The Vestfold Cardiac Arrest Registry in Norway contains all the operational and clinical data from prehospital cardiac arrests in Vestfold County from 2007 to 2020 and Telemark County from 2007 to 2015. Data from LIFEPAK 12/15 defibrillators (Stryker, Redmond, WA, USA) were stored in Vestfold and Telemark CODE-STAT data review software system (Stryker, Redmond, WA, USA), at Vestfold Hospital Trust. The two registries were approved by the respective ethical committees and accordingly every patient with a sufficient neurologic recovery signed an informed consent. The Vestfold Cardiac Arrest Registry dataset originates from the standard electronic records in the Vestfold and Telemark Emergency Medical Communication Centre (EMCC Vestfold and Telemark). The electronic ambulance records from all ambulance missions in those counties were stored in the same system (AMIS database). The Medical director for the EMS (JESH) revised all cardiac arrest records. A report made in Crystal Reports extract the relevant clinical and operational data from the AMIS –database and generates the Vestfold Cardiac arrest Registry, which in this case are in Excel-format and stored on a secure server dedicated for research. One of the authors (JESH) manually anonymised the registry by deleting all columns and cells containing personal data. An equivalent process was done in the Code Stat files.

**Emergency medical systems**

The province of Pavia covers a rural and urban area of 2.969 Km^2^ with a population of about 535.000. A national emergency telephone number 112 is connected to the regional dispatcher center. The province is served by six vehicles with advanced life support (ALS)-trained staff [equipped with a Corpuls 3 monitor/defibrillator, a physician and a specialized nurse, or by a specialized nurse only] and by 33 ambulances providing basic life support – defibrillation (BLS-D). In addition, five helicopters with a physician and a specialized nurse which serve the entire region of Lombardy may be dispatched. In case of suspected OHCA, the EMS dispatcher activates one to three emergency vehicles of which at least one has a physician on board. The EMS dispatcher also provides telephone CPR to the caller. If the first vehicle arriving at the scene is staffed with BLS-D-trained personnel, the rescuers are instructed to initiated resuscitation (including CPR and use of an automated external defibrillator [AED]) unless clear signs of death are present (rigor mortis, hypostasis, injuries not compatible with life).

Vestfold and Telemark Counties in the Southeast of Norway are also a mixed rural/ urban area of 15102 km² with an average population about 380 000. All the medical emergency calls from both counties to the Medical Emergency Telephone number 113 are received at the Emergency Medical Communication Centre (EMCC) at Vestfold Hospital Trust in Tønsberg. The EMS call taker/medical operator provides telephone CPR to the caller or supervises already ongoing CPR unless clear signs of death are present. The EMS is hospital-organized under the Vestfold Hospital Trust in Tønsberg and Telemark Hospital Trust in Skien. There are 18 ambulance stations with 1-4 vehicles. The ambulances are staffed with two paramedics, of which at least one is ALS-certified. The EMCC usually dispatches two ambulances for prehospital cardiac arrest, and when available a doctor-staffed air ambulance or ad hoc an anaesthesiologist in a third vehicle from the closest hospital. In the most rural areas, a general practitioner on duty may act as a part of the team.

**Sample size**

Because of the lack of evidence on this topic, the expected reduction of AMSA due to amiodarone administration is unknown. We powered the study to be able to detect even a difference of 1 mV · Hz with a standard deviation of 6 mV · Hz in both groups. Based on this, and considering that the average number of shocks delivered to patients receiving amiodarone was double than those delivered to non-amiodarone patients, assuming a type I error (α) of 0.05 and Type II error (β) of 0.2, a total of 1300 shocks were needed.
