# Supplementary figures and images for: aMplitude spectral area of ventricular fibrillation and amiOdarone Study in patients with out-of-hospital cArdIaC arrest. The MOSAIC study
Source: Front Cardiovasc Med. 2023 May 15;10:1179815. doi: 10.3389/fcvm.2023.1179815 (PMC10226588; doi:10.3389/fcvm.2023.1179815)

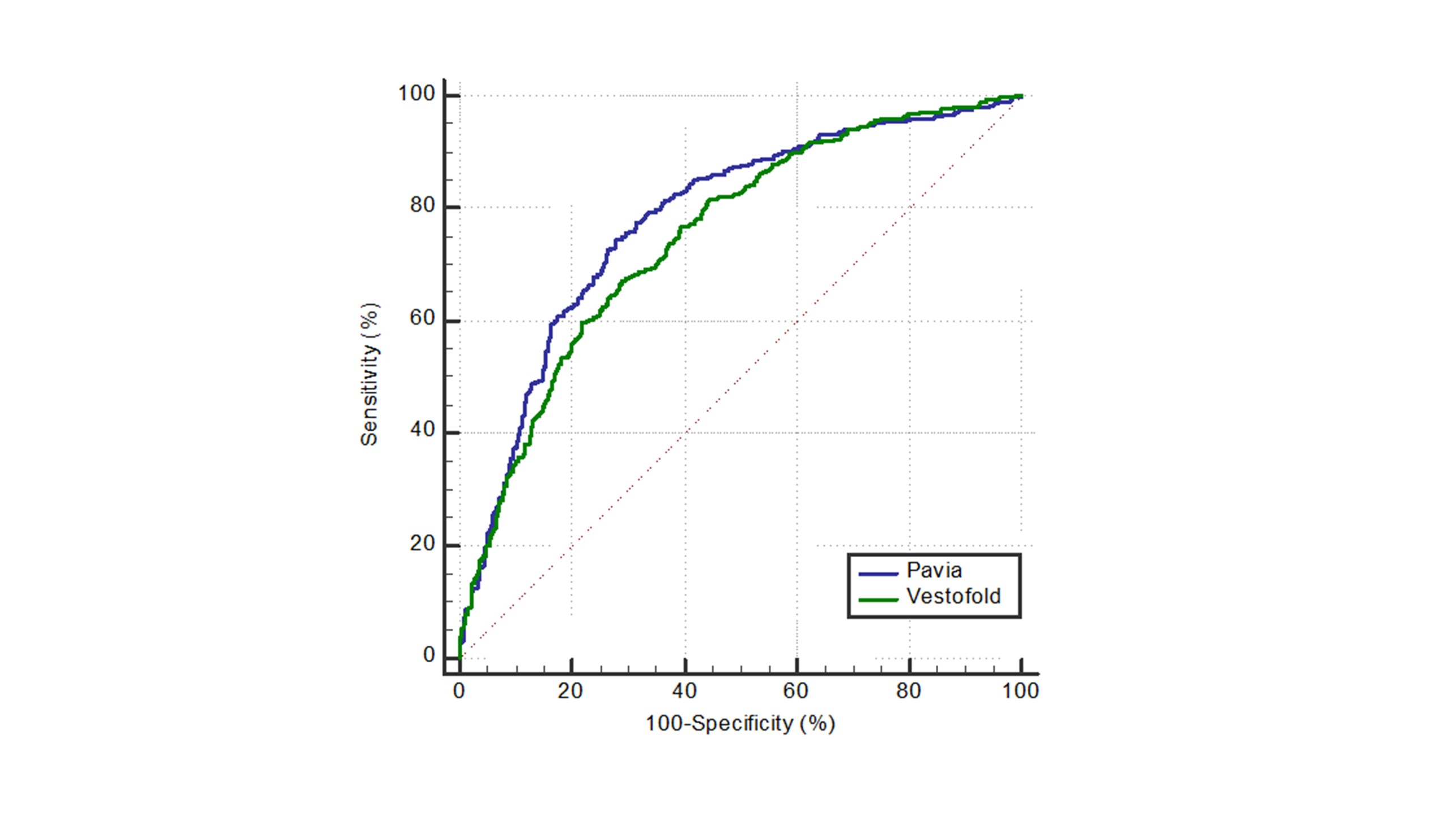

Supplement: Supplementary file 3 [file Image1.jpeg]
